# Case Report: Isolated Idiopathic Saccular Dysfunction

**DOI:** 10.3389/fneur.2021.753433

**Published:** 2021-11-15

**Authors:** Sofia Waissbluth, Javier Oyarzún

**Affiliations:** Department of Otolaryngology, Pontificia Universidad Católica de Chile, Santiago, Chile

**Keywords:** otolith, saccule, vertigo, dizziness, case report

## Abstract

Advances in vestibular testing have now allowed us to test each semicircular canal as well as the utricle and saccule, independently. This has led to the discovery of new patterns of vestibular dysfunction that were once impossible to evaluate. This report describes the case of a 20-year-old woman with a 2-month history of recurrent dizziness. She had a complete audiovestibular assessment. The only abnormality observed was the absence of a cervical vestibular-evoked myogenic potential response for the right side, hence an isolated saccular dysfunction. In conclusion, isolated otolithic dysfunction is probably an overlooked and neglected clinical presentation. Its true incidence is unknown, and further research is needed to understand this clinical entity.

## Introduction

Vestibular testing has evolved tremendously in recent years, and with the introduction of the video head impulse test (V-HIT), and the cervical (cVEMP) and ocular vestibular-evoked myogenic potential (oVEMP), it is now possible to evaluate the semicircular canals (SSC), as well as the saccule and utricle, independently. Prior available tests include the caloric test and rotational chair, which allowed the evaluation of the horizontal SSC function at different frequencies (1). However, dynamic otolithic function can be impaired independently from the horizontal SSC and would otherwise be unknown without the recent introduction of VEMPS (2).

While the superior vestibular nerve has afferents from the horizontal SSC, anterior SSC, the utricular macula, and a small contingent portion of the saccular macula, the inferior vestibular nerve has fibers from the posterior SSC and the saccular macula. Because of VEMPs and vHIT, it is now possible to diagnose superior or inferior vestibular neuritis more accurately. These tests have also been an incredible tool in clarifying conditions with selective end-organ impairments (1). While acute vestibular syndrome is a common cause of otoneurological consultation, other patients have non-specific symptoms that do not necessarily fall into a clear diagnosis.

We hereby present the case of a young female patient with unilateral saccular pathway impairment, with otherwise normal audiovestibular testing.

## Case Description

A 20-year-old female patient was seen at the otorhinolaryngology department. She presented with a 2-month history of mild recurrent spontaneous dizziness that lasted a few seconds, many times a day and occurred every day. She also reported mild dizziness a few minutes after lying down, this was not frequent. It was not positional dizziness, it occurred ~5 min after going from a sitting to a supine position. She did not feel any unsteadiness, vertigo, floating sensation, swaying, rocking, or lateral translation. She did not have any auditory symptoms (aural fullness, fluctuating hearing loss, hearing loss, tinnitus, otalgia, otorrhea). She did not have any history of migraines nor drop attacks or headaches. She did present with a vestibular neuritis of the left side 6 years prior. Her physical exam, including an otoneurological assessment, was unremarkable. Romberg and Unterberger's stepping test were normal.

She underwent a complete audiovestibular evaluation including acoustic immittance testing, audiometry, videonystagmography, V-HIT (Otometrics ICS Impulse), oVEMP, and cVEMP (Eclipse, Interacoustics). For V-HIT, the right eye was recorded and all three canals were evaluated. The oVEMP and cVEMP were performed using an in-ear earphone, with 500 Hz tone-burst stimulation delivered at 100 dB nHL (123.5 dB peSPL); stimulation rate of 5.1 Hz; and 500 sweeps for oVEMP and 200 sweeps for cVEMP for each testing session. The equipment for cVEMP testing includes electromyography monitored stimulus and recording as well as scaling to guarantee adequate sternocleidomastoid muscle contraction and comparable results. In this case, the patient had EMG values above 50: 84.8 μV on the right side and 109.9 μV on the left side, hence adequate muscle contraction.

She also had a computed tomography (CT) scan of the temporal bone as well as a magnetic resonance imaging (MRI) of the brain with emphasis on the posterior fossa.

**Figure 1:**
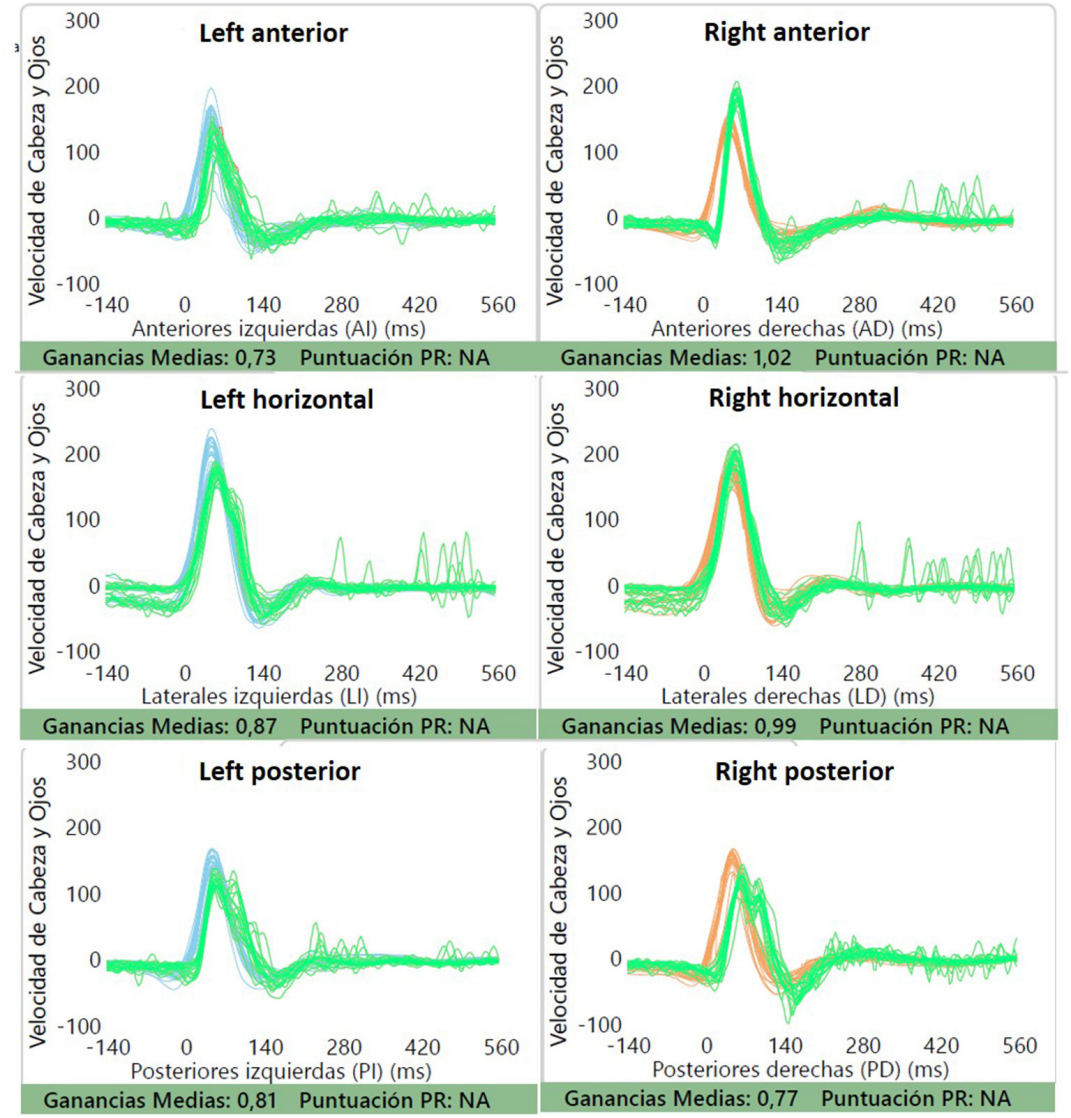
V-HIT testing was normal, with normal VOR gains for all canals and absence of saccades. Gains can be seen for each canal.

**Figure 2:**
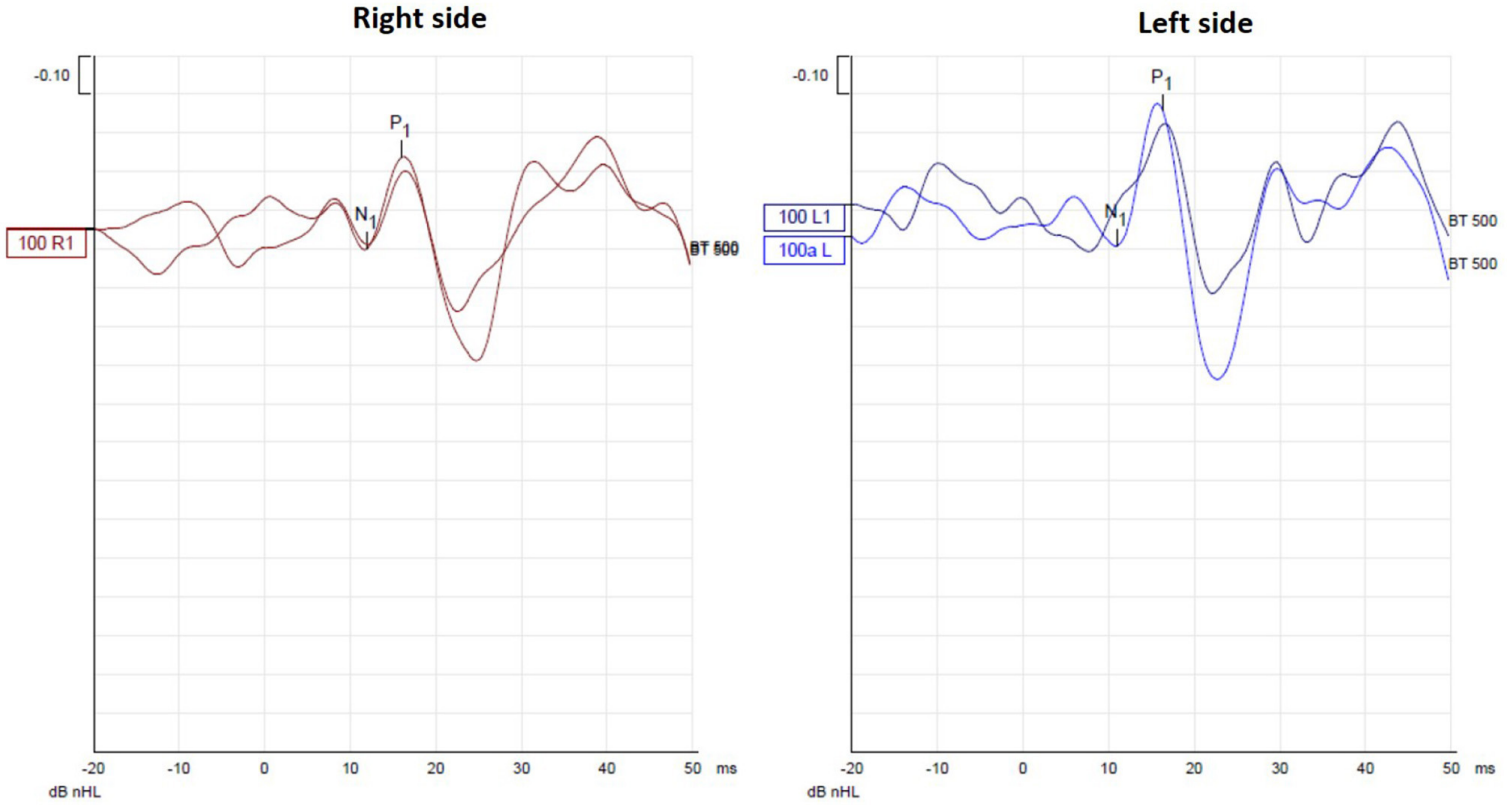
oVEMP testing did not show a significant difference in latency and peak-peak amplitude of N1-P1 between both sides, with a 19% asymmetry ratio.

**Figure 3:**
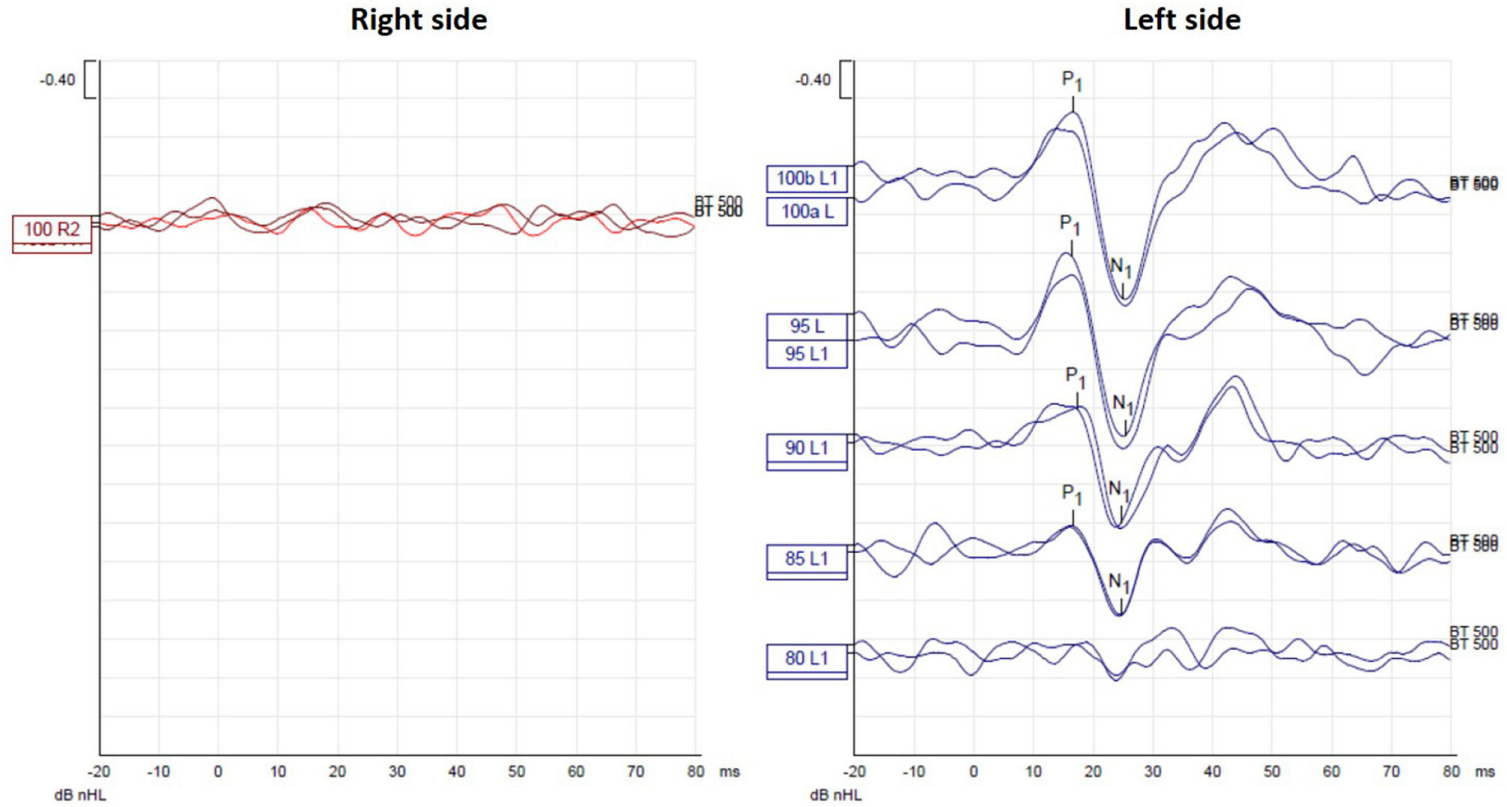
cVEMP testing showed absence of P1-N1 complex for the right side, while it was present for the left side.

Her hearing was within normal limits with bilaterally symmetrical audiometric curves, type A tympanograms, and conserved acoustic reflexes. She did not exhibit spontaneous or positional nystagmus. The V-HIT test showed VOR gains within normal limits for all canals, without any overt or covert saccades (Figure 1). The oVEMP did not show a significant difference in latency and peak-peak amplitude of N1-P1 between both sides, with a 19% asymmetry ratio (Figure 2). This asymmetry is not significant as the normal reference interval according to our own institutional trials is 33%. For the cVEMP, however, no P1-N1 complex could be elicited for the right side, while it was present for the left side (Figure 3). This pattern was confirmed with test-rest on one occasion. Unfortunately, we have no repeated measures, so we cannot attain whether this VEMP response was persistent or only occasional. The CT scan of the temporal bone was normal, without any signs of third window lesions. The brain MRI was normal.

On the latest follow-up, 1 month after the initial consult, the patient did not report any symptoms and she did not want to pursue any further testing and did not feel it was necessary to pursue vestibular rehabilitation.

## Discussion

Patients with a history of dizziness can now be assessed for all peripheral vestibular organs, independently. In this case, the patient presented with a 2-month history of non-specific dizziness. It did not impact her daily life. Because she had previously experienced vestibular neuritis years prior (contralateral side; acute vestibular syndrome with unilateral hypofunction on caloric testing at that time consistent with vestibular neuritis; no VEMP testing was performed then), she was keen to see an otorhinolaryngologist. Her only abnormal finding was an absent P1-N1 complex for the cVEMP on the right side and, at a 1-month follow-up, did not report any specific symptoms. The patient appears to have an isolated saccular impairment.

More is known about isolated utricular dysfunction. Perhaps because the superior vestibular nerve receives afferents from the horizontal canal, and there is vast experience with vestibular neuritis which mostly affects the superior branch, there has been more investigative efforts in this area. Also, because benign paroxysmal positional vertigo (BPPV) is the most commonly diagnosed peripheral vestibular disorder, and it is believed that utricular dysfunction could lead to otoconial detachment and consequently, BPPV (3). In a recent article by Fujimoto et al. (2), evaluating 76 patients with normal caloric and V-HIT, but abnormal responses in cVEMP and/or oVEMP, the most common diagnosis was BPPV. Pelosi et al. (4) also performed a retrospective analysis of patients with vestibular complaints and abnormal or absent oVEMP responses. They observed that the most commonly associated diagnoses were migraine and benign positional vertigo. Reported symptoms included non-vertiginous dizziness, vertigo, and postural instability; however, 81% also exhibited otologic symptoms. They do not, however, discuss audiometric results or whether patients actually had sensorineural hearing loss. Hence, the abnormal oVEMP responses may not have been isolated with regards to hearing loss.

Murofushi et al. (5) describe that patients with episodic lateral tilt sensation without any other vestibular symptoms and normal caloric testing displayed abnormal otolith-ocular reflexes as evidenced by oVEMP testing, suggesting that these patients were suffering from utricular dysfunction. However, of the 10 patients with abnormal oVEMP responses, 5 also had abnormal cVEMP responses. Also, V-HIT testing was not performed. In another study, Murofushi et al. (6) evaluated patients experiencing episodic tilting or translational sensations in the pitch plane. Of the 11 patients included, 4 has absent or decreased cVEMP responses and normal oVEMP and caloric testing, and the duration of the episodes lasted from a few minutes to one day.

Recently, a proposal on the diagnostic criteria of definite isolated otolith dysfunction was published. The aim of that study was to compare the differences in the clinical presentations between cases that had isolated otolith dysfunction, with and without symptoms (7). In the group with symptoms, only 2 out of 11 had unilateral saccular involvement, and none had spinning vertigo. Fujimoto, on the other hand, reports six cases of isolated unilateral saccular dysfunction; five had rotatory vertigo (2). Because the saccule is a sensor of linear acceleration, and the cVEMP tests mainly dynamic saccular function (8), the tendency would be to assume that patients with isolated cVEMP abnormalities would present with non-spinning vertigo (7).

The questions that remain unanswered are (1) whether the symptoms are actually due to the saccular dysfunction, (2) whether the saccular involvement is isolated or is an initial stage of a future vestibular disorder such as an initial stage in Ménière's disease or vestibular migraine, or (3) if the absent cVEMP response is due, in fact, to saccular involvement seen as the cVEMP test evaluates the vestibulocollic reflex pathway. Chua et al. (9, 10) most recently published their experience with isolated otolithic dysfunction in a tertiary care center. The most common clinical finding was “no clear diagnosis” (65.5%) followed by vestibular migraine (13.6%). They conclude that while laboratory test results are important, the patients' subjective complains and functional impairments should be prioritized when planning rehabilitation strategies to aid in daily life activities. In our case, the patient did not fulfill the criteria for vestibular migraine or any other specific otoneurological problem, and the dizziness did not affect her quality of life. An important point to keep in mind is the fact that we do not have repeat cVEMP testing at a longer follow-up period when the patient was not symptomatic, which may have shown a similar or modified patterns of response. This area of research requires further investigation.

VEMP testing has shown good test-retest reliability and shows a robust response in young individuals (11). Important aspects to remember for cVEMP testing include the patient's age, as older patients may have absent or reduced responses, and also recording with tonic contraction of the sternocleidomastoid muscle (12).

## Conclusion

Isolated saccular dysfunction may be a cause of dizziness that may be overlooked if cVEMP is not performed when assessing a dizzy patient with normal semicircular canal function. Further research is needed in order to better understand isolated otolithic dysfunction.

## Data Availability Statement

The original contributions presented in the study are included in the article/supplementary material, further inquiries can be directed to the corresponding author/s.

## Ethics Statement

The studies involving human participants were reviewed and approved by Comité Ético Científico de Ciencias de la Salud, #210601002. The patients/participants provided their written informed consent to participate in this study.

## Author Contributions

All authors listed have made a substantial, direct and intellectual contribution to the work, and approved it for publication.

## Funding

This project was funded by Fondecyt 11201142 for SW.

## Conflict of Interest

The authors declare that the research was conducted in the absence of any commercial or financial relationships that could be construed as a potential conflict of interest.

## Publisher's Note

All claims expressed in this article are solely those of the authors and do not necessarily represent those of their affiliated organizations, or those of the publisher, the editors and the reviewers. Any product that may be evaluated in this article, or claim that may be made by its manufacturer, is not guaranteed or endorsed by the publisher.
